# Abstracts and Gleanings

**Published:** 1881-10-20

**Authors:** 


### ABSTRACTS AND GLEANINGS.

**Otorrhœa.**—Since the results of a chronic purulent otitis media are or may be so appalling, the question of the proper treatment becomes a very urgent and important one, and it will not do for any physician into whose hands a family have intrusted themselves in good faith, relying upon his knowledge of disease, to say, as I have too often heard, "Well! I don't know anything about ear diseases, and I don't want to."

This disease is usually very easily diagnosticated and the rationale of the treament is very simple : cleanse the ear thoroughly with syringe and absorbent cotton, and inspect it through a speculum with reflected light. A speculum may be bought for fifty cents, and in these days every physician should have a laryngoscopic mirror, but if you haven't, take a bit of looking-glass and scrape a little hole through the silver. With this simple reflector you can light up the meatus so that you can tell whether its walls look healthy or not, and if they do, you can reason by exclusion that the middle ear is affected, though you do not get a satisfactory view of the drum membrane.

Usually a hole in the ear drum can be seen and distinguished, but often it cannot. It may be in the lower anterior segment, out of sight, around the bend in the external meatus; or the drum membrane may be inflamed and discolored so that you are not sure whether it is drum membrane which you see or the internal wall covered with mucous membrane. Sometimes you can diagnosticate a perforation by listening closely while the patient inflates the Eustachian tube and middle ear by the Valsalvian method. If a perforation exists and the Eustachian tube is pervious, you will hear the whistle of the air as it passes through. I have read somewhere of putting a light bit of cotton in the ear in such cases and expecting to see it blown out, but this experiment has never succeeded under my observation. If you cannot hear the whistle of air, and are still in doubt, drop a little warm water into the ear and watch it through the speculum while the patient inflates the ear, and you may be rewarded by seeing bubbles of air break through the water. Of course, if you have a Politzer bag you won't linger over the Valsavian method, and one pull through a Siemen's speculum settles the cases, but I am not supposing that you have an Otologist's armament.

Having established your diagnosis, the treatment comes next. You want to restore that mucous membrane to its normal condition, where it is possible, and induce a cicatrization of ulcerated surfaces. The first need is cleanliness, and cleanliness without violence. For this you will need more tools. A mirror on a head band, which leaves both hands free, is absolutely essential; no one has any business with a purulent otitis media without one. After syringing the ear long and carefully, dry it out with absorbent cotton, under your eye. You must always look and see where you are going, the structure of the ear is too delicate for blind poking. Patients and friends cannot cleanse an ear, you must do it yourself, or it won't be done. I know one man who after fifteen or twenty years' practice can cleanse his own ear. He puts a piece of slender flexible rubber tubing on the nozzle of his syringe and inserts that into his ear; as he has no ear drum, the way is clear to the bottom of the hole. After pumping from a pint to a quart of water through this, he dries his ear with absorbent cotton on a cotton holder, and this he can insert clear into the middle ear. As he does this every day, no pus collects and hardens. This is the single exception that proves the rule that no man can cleanse his own ear.

After the ear is clean, the question of what application to make comes up, and here a pretty wide choice presents itself, and the very variety of the remedies which have been mentioned only proves that no one has been found entirely satisfactory. I more frequently begin with blowing in finely powdered alum than in any other way, and repeat this every other day. I often, when this fails or seems to have too little effect upon the inflamed membrane, use finely-powdered iodoform or iodoform and alum. The great objection to iodoform is its odor, and this becomes very disagreeable in using iodoform in this manner. When one puffs a cloud of iodoform in a patient's ear, the return blast loads his mustache with the powder, and he carries it under his nose the rest of the day.

Some medical journals, a few months ago, vaunted boracic acid as the application in purulent otitis media, but in my hands it has not proved a specific by any means, but a useful variation. These powders act better when the drum membrane is almost entirely destroyed, I think, and a puff applies them thoroughly to every part. When the perforation is small and in the upper part of the membrane, the case presents peculiar difficulties. It is very difficult to cleanse such an ear, and here especially, after syringing thoroughly, it is beneficial to blow through the Eustachian tube by Valsalva's method, or the air bag, to force the middle ear fluids through the perforation in the external meatus, where they can be wiped out with cotton. In such cases I have had the best results from the use of strong solutions of nitrate of silver .10 to .15.

After cleansing the ear by all ways as thoroughly as possible, I have the patient lie on the side, with the affected ear uppermost. Then with a pipette introduced well into the ear, I drop in 10 to 15 drops. I then pull and work the ear so as to churn it down clear into the middle ear, and be sure that it reaches every part. After leaving it a few minutes, I draw out what I can with the pipette, and put in a little pure warm water, then pump this out and put in some more, finally I drop in a solution of salt to decompose the nitrate of silver that may be left. The principal object of this procedure is cosmetic, Should the patient arise with an ear full of a .15 solution of nitrate of silver, a long, dirty-brown streak from ear to shirt collar would confront you at the next visit. Sometimes you have the good fortune to see a perforation close up, which is a consummation most devoutly to be wished, as this protects the mucous membrane of the middle ear from external irritants. If the drnm membrane does not close up and the secretion stops, it is best to wear a loose fledget of cotton for a protection.

Dr. Howe spoke very highly of permanganate of potash in solution, for purulent otitis media, in a paper published some time ago. He gave it to the patients for ear drops. I used it some, but the patients objected to the stains left by this salt on everything it touched, and in my hands it did not prove as efficacious as I had hoped it might from the statistics given in its favor.

Where patients cannot come to the physician for treatment, they should provide themselves with a good ear syringe and keep the ear as clean as possible. If all the pus and mucus is not removed, it can be kept fluid and wholesome, so that none will be blocked in behind dry hardened masses, and the penetrating odor, which makes so many running ears an offense to the whole household, will be prevented. Should mastoid-cell complications arise, an incision should be made into the mastoid at once with strong knife or trephine.—Dr. Abbott, in *Buff. Med. and Surg. Journal.*

**The Uuse of Quebracho in Dyspnœa.**——Dr. Andrew H. Smith, chairman of the Committee on Restoratives of the Therapeutical Society of New York, has submitted, on behalf of the committee, a report, founded on clinical data, on the use of quebracho in dyspnœa, which is published in the "New York Medical Journal and Obstetrical Review" for September, 1881. Of the thirty-two cases covered by the report, eleven were of spasmodic asthma, with or without emphysema and bronchitis. Of these, in nine cases the dyspnœa was notably relieved. In two cases of asthma associated with bronchitis no benefit resulted. One patient with emphysema and bronchitis without asthma was relieved. One with bronchitis with obesity was not relieved. Two with mitral insufficiency were not relieved. One with mitral stenosis was not relieved. One with hypertrophy with dilatation was not relieved. In two cases of cardiac disease (form not stated) the dyspnœa was relieved. In one case of fatty heart there was slight relief. Two patients with dyspnœa depending upon Bright's disease, in one of whom pulmonary œdema was noted, were relieved. In one case of aortic aneurism the dyspnœa was relieved till near the close. In one case of tonsillitis the dyspnœa, partly nervous, was relieved. In one case of cancer of the lung dyspnœa was relieved. In two cases of pneumonia it was relieved. One patient with hysterical dyspnœa was relieved. In one case of catarrhal phthisis, second stage, the dyspnœa was relieved. In one case of catarrhal phthisis, third stage, it was not relieved. In one case of intermittent fever with old pleurisy, the patient being an opium-eater, the dyspnœa was increased. Thus the thirty-two cases of different diseases in which dyspnœa formed a prominent feature, this symptom was relieved to a greater or less extent to twenty-one; not relieved in ten: aggravated in one. In some instances the treatment was not pushed far enough to give a decisive result. It is possible that the nausea observed in some cases might have been avoided by the use of smaller doses, and perhaps a favorable result obtained. The fact that dyspnœa depending upon such a variety of causes may be relieved by quebracho points, says the writer, to the respiratory center as the seat of its action. Apparently it blunts the sense of want of air, and thus mitigates the suffering from a deficient supply. But this action is not necessarily only palliative. Exaggerated respiratory efforts are often in themselves an evil, not only on account of the muscular effort expended, but from the aspiration of blood into the thoracic viscera, which results especially when the dyspnœa is caused by narrowing of the air-passages rather than by solidification or compression of the lung. Hence in many cases an agent which will moderate the violence of the respiratory movements will not only lessen the distress of the sufferer, but will increase the chances of his recovery. That quebracho will often very promptly fulfill this indication there seems to be no room to doubt, while as yet there is no evidence that it is liable to produce unfavorable after-effects. The extremely disagreeable taste of the medicine and its tendency to produce nausea are, however, serious drawbacks to its use by the mouth. As yet, we have no record of its employment by the rectum. If the active principle is isolated, so that it can be used hypodermically, a great advantage will have been obtained.—*Va. Med. Monthly.*

**Aspiration of the Gall-Bladder.**—Dr. P. H. Kretzschman reports a successful case in the Proceedings of the Medical Society of the County of Kings, September, 1881, of which he says:

Five times has the gall-bladder been aspirated; thirty-four ounces and a half of bile have been removed within one month. At every operation the patient felt much relieved, and since the first withdrawal of bile the constitutional symptoms diminished in severity. At no time did the operation itself place our patient in danger, and, generally speaking, there was no pain attached to it.

The following generalizations he appends to his paper:

1. The operation can be performed with safety without taking particular precautions in uniting the walls of the gall-bladder with those of the abdomen.

2. The operation can, therefore, be done as soon as the diagnosis of a dilated gall bladder has been made, if, from its size, there seems to be danger of rupture, or if the patient suffers much pain. Aside from these conditions, when aspiration should be resorted to without hesitation, the question presents itself whether it would not be good practice to evacuate the contents of a distended gall-bladder under all circumstances, simply to remove the superfluous bile, which, being cut off from its natural destination, is bound to be re-absorbed by the lymphatics, carried back into the circulation and produce, to a greater or less degree, a condition which is generally known as "cholæmia."

3. A very fine trocar, such as would not be of much value, in case of simple puncture, can be employed, and by means of suction even a tenacious fluid can be removed from the gall-bladder.

4. The insertion of a small trocar or an aspirating needle is almost a painless procedure.

5. In case of doubt as to the presence of gall-stones, a flexible probe can be passed through the canula and used as a sound.

6. Aspiration being a safe and painless operation, it can be employed for the purpose of aiding diagnosis.

The rules for performing the operation are thus formulated:

1. Aspiration should not be delayed, but resorted to as soon as the diagnosis of distended gall-bladder has been made.

2. A good-sized aspirating needle or a fine trocar should be used.

3. The instrument should be introduced into the gall-bladder at a point as high up and as near to the border of the liver as possible.

4. On withdrawing the instrument, the punctured wound in the abdominal wall should at once be closed by some kind of a plaster, or by the introduction of a stitch.

5. The operation should be repeated as often as the gall-bladder becomes distended again,

6. The common rules of surgery, as to cleanliness, etc., should be strictly adhered to.—*Medical Herald.*

**The Treatment of Ranula.**—An important discussion took place before the Societe de Chirurgie on the treatment of ranula, in which nearly all the members took part. M. Deleus recited a case in which the cyst was excised and cauterized, but at the end of two months it returned. This fact, he believed, resulted from the migration of the sub-lingual ranula through the muscular fibres of the floor of the mouth and developing a cyst in the buccal cavity.

M. Trelat for many years excised with the scissors in the case of small ranulæ, and when they were more voluminous he treated them by puncture and the injection of iodine.

M. Despres treated every kind of ranula by the drainage, and always with success.

M. Verneuil observed that he tried many methods in the treatment of ranula, but with varied success. He adopted the plan of slow section, for which purpose he passed a curved needle charged with a double thread of silver wire through the cyst, and united both ends in a firm knot. In five or six days the section was effected.

M. Labbe did not doubt the success obtained by M. Despres by his method, but he considered that to keep a seton in the mouth for six months to cure a ranula constituted a veritable infirmity.

M. Despres, in replying, said that he never knew a patient to complain of it.

M. Le Dentu said that M. Auger employs the injection of two drops of chloride of zinc in the deliquescent state into the cyst without previously evacuating it. This treatment always succeeds; there follows a sharp inflammatory reaction, but it is by no means dangerous. The inflammation subsides in five or six days, and at the end of ten days the cure is complete. For small ranulæ one drop of the liquid suffices, and if the cyst is very voluminous it is preferable to draw off a little of the contents before introducing the chloride of zinc.

M. Gilette could not agree with M. Le Dentu in considering that chloride of zinc was not attended with danger and that it was always successful. He had seen M. Auger at the Hopital Beaujon inject three drops, and the pain was so intense that the patient tried to jump out of the window; and after all the cyst returned and was eventually excised.—*Med. Press and Circular.*

**Treatment of Chorea.**—At the end of a paper on chorea, based upon an experience of one hundred cases, Dr. William Strange speaks of the treatment, saying that the changes must be rung on the so-called nervine tonics, varying them according to the temperament of the child or to the collateral symptoms accompanying the choreic movements. If pallor, palpitations, and loss of weight exist, iron or arsenic, or both, will be necessary. If, on the contrary, the vascular system be sufficiently full and the motile element prevails, then the bromides with ammonia, or the succus conii will be of most avail. Frequently, whatever the condition of the vascular system and of the general nutrition, no good arrives until we have succeeded, by sedatives, in calming the excessive mobility of the nervous system. In these cases Dr. Strange has used the ice-bag to the spine and the ether spray to the nape of the neck, but not with much success. Direct calmatives—digitalis, belladonna, cannabis indica, with the bromides —answer the best.

The nervous symptoms once quieted, iron or arsenic may now be given, and carried to a somewhat high degree. Some have recommended large doses of arsenic, ten to fifteen minims of Fowler's solution; but Dr. Strange has seldom found that the stomach will tolerate these large doses, and has contented himself with much smaller ones, in combination with iron or zinc.

But, whatever the remedy selected, it will be necessary to continue its administration until it has produced its special physiological effect. Especially is this necessary with the neurotic sedatives. Children bear large doses of belladonna and conium; and Dr. Strange has never found this class of remedies do much good until these full physiological effects (consistent with safety) have been produced.

Dr. Strange used some years ago to treat all his cases of chorea with wine alone, the port wine of the hospital, merely clearing out the primæ viæ, to make sure that trouble was not caused by entozoa or depraved alvine secretions. The amount given was three to six ounces daily, and all the cases got well. After suspending this treatment for some years, he has recently recommenced it with good results.—*Med. Times.*

**Medical Thermometry.**—This subject has come to be regarded as one of very great importance, both in medical and surgical practice. The ordinary means of determining the temperature of the body are so very imperfect, that a considerable deviation may be present and escape observation. All abnormal temperatures denote the presence of disease, and in many cases the physician is greatly aided in his diagnosis and prognosis of a case by ascertaining the temperature of the body, and this, with the means at our command, may be determined with a nicety which is common to few other phenomena. The temperature of the body cannot be feigned or falsified, and its abnormality may decide the degree or danger of the attack. Relapses, complications, or transitions in the course of disease, may, in this way, be discovered before they could otherwise be recognized. It reveals the imminence of a fatal termination, or the impossibility of recovery. In surgery the application of the thermometer determines the practicability or possibility of an operation where there are grave doubts.

The variations of temperature, however, to be of any real practical value must be taken regularly night and morning. A single observation, while it may point out that a patient is very ill, is not by itself conclusive as to the kind of disease present. When we have extremes of temperature we know there is great danger. For example, temperatures below 96.6F. are *collapse* temperatures, 92. fatal collapse. Temperatures from 100 in the morning to 104 in the evening, are *febrile* temperatures, and temperatures from 104 in the morning to 107 in the evening are *hyperpyretic* temperatures, 107 and above indicating a fatal termination. Every medical practitioner is aware of the difficulty in diagnosing at an early stage between the different febrile diseases, and it is here that the thermometer comes largely to our aid. If temperature is normal, or only slightly increased, pneumonia, scarlet fever, typhoid fever and small pox are excluded; if the temperature is high at the outset, typhoid fever, influenza, and articular rheumatism, pleurisy, intermittent and ephemeral fevers, the exanthemata, pyæmia, or meningitis, may be present.—*Canada Lancet.*

**Radical Treatment of Hydrocele by Injection of Carbolic Acid.**—At a meeting of the Philadelphia Academy of Surgery June 7, 1880, R. J. Lewis stated that in 1879 he had begun to treat hydrocele by carbolic acid injections, because a more plastic grade of inflammation than that obtained by ordinary injections was required, and because incision only accomplished a cure through suppuration. His method is to withdraw the fluid by an ordinary trocar, and then introduce the long nozzle of a syringe through the trocar into the vaginal sac. By this means the carbolic acid is thrown into the cavity, and there is no danger of its being injected into the cellular tissue of the scrotum. The carbolic acid crystals are merely liquefied by slight heat, or by a few drops of glycerine. To keep the injecting fluid ready for use at all states of temperature, about ten per cent of glycerine or water may be added to the crystals. The amount of carbolic acid which Dr. Lewis injects is one-half a fluid drachm, and this is allowed to remain in the vaginal tunic.

The operation is almost, if not entirely, painless, because of the local anæsthetic action of carbolic acid. The patient sometimes exclaims at the moment of introduction, but has a sensation of numbness rather than of pain. The pain, when tincture of iodine is employed, is much greater. Care should be observed to allow no acid to flow upon the external surface of the scrotum, for pain and inflammation will follow such contact.

After the injection the patient is permitted to walk about the house until the weight and slight soreness of the scrotum cause him to lie upon a bed or lounge. The results of this method of treatment are excellent, for undue inflammation does not occur, there is no marked pain, and a radical cure generally ensues. Dr. Lewis has never seen suppuration or sloughing follow this manner of dealing with hydrocele. —*Phila. Mdd, Times.*

**Two Yards of Intestine Removed.**—A Paris letter in an English contemporary describes a remarkable case in the practice of Dr. Kœberle, of Strasburg:

The patient, a young woman, aged 22, had been subject for some years to acute attacks of abdominal pain, and on two occasions there had been symptoms of obstruction, which had, however, been overcome by the use of enemata. Since that time (October, 1880) the pain had been most constant and severe, not remitting day or night, and at times so intense that it could scarcely be soothed by hypodermic injections of morphia. Gastrotomy was performed on November 27th, 1880, and four narrowings were found in the bowels, one being only four millimeters in width. These strictures were distributed over two yards of gut (2 meters .05), which was excised between two ligatures at each extremity, the vessels of the mesentery being secured by twelve ligatures. The ligatures of the ends of the intestine were then tied together so as to place the gut in the most favorable position for enterotomy, which was performed on the third day. The parts beyond the ligatures came away between the 12th and 15th days, and on the 20th the first alvine evacuation occurred. Five days later a band of strapping sufficed to prevent food or gas passing through the wound, which had entirely healed in six weeks. The operation was not performed antiseptically, and the temperature never rose beyond 38° Centigrade. During convalescence, and from the third day, the patient was fed by the mouth, with solid and substantial food—bread, meat and eggs—sufficient liquid being given for the purpose of digestion only, the thirst being assuaged by injections of water in the rectum, seventy such injections having been made in the twenty days. The young woman, who is now quite well, has no pain or digestive trouble of any kind.—*Med. and Surg. Rep.*

**Treatment of Eczema of the Hand.**—A case of this refractory disease is reported in the Dublin Medical Journal, by Dr. A. W. Foot:

The patient was twenty-two years old. The disease was confined to the back of the hand and the clefts between the fingers, where were many fissures, the viscid secretion issuing from which formed crusts with a pustular aspect. The pruritus was very severe.

Each finger and the entire hand were wrapped round with strips of old linen soaked in a mixture of lead lotion and glycerine, and the whole then sealed up in gutta-percha paper. As the itching had quite broken her sleep at night, she had, for two or three nights, taken draughts with potassium bromide ʒss and chloral 15 grs., in chloroform water, and she was ordered 5*m* of Fowler's solution in tincture of bark three times a day after meals. As there was no reason to starve her, she was given meat and porter every day.

The inflammatory action was soon moderated by the lotion, which was applied fresh every day, and the hand sealed up again after having had a jug of cold water poured over it. It was kept in a sling; the perfect rest obtained by slinging the muffled hand, and the exclusion of the air by the careful sealing up of the gutta-percha cover, are points to be attended to. Whenever the hand was let hang or rest in her lap, it got hot, heavy and swollen, and began to throb. After three days of this treatment the heat, redness and itching had abated; then a thirty grain solution of nitrate of silver was carefully painted all over the back of the hand and fingers, from the wrist to the margin of the nails, avoiding the latter, and sealing up and slinging continued. In a few days she got a strong lotion of iodide of potassium to remove the blackening effects of the nitrate of silver, which it is quickly doing, and the sealing of the hand was discontinued. The arsenic had to be omitted for a few days in consequence of gastric irritation. The note of June 8th (she was admitted May 26th), is—she "has great use of the hand," and it is white, dry, and quite free from itching.—*Med. and Surg. Rep.*

**Poisoning by Strychnia.**—Dr. Bartholow, in New York Medical Record, says:

1. That after a fatal dose of strychnia, life may be saved by bringing the animal under the influence of chloral hydrate.

2. That chloral hydrate is more likely to save life after a fatal dose of strychnia than strychnia is to save life after a fatal dose of chloral hydrate.

3. That after a dose of strychnia, producing severe tetanic convulsions, these convulsions may be much reduced, both in force and frequency, by the use of chloral hydrate, and consequently, much suffering saved.

4. That the extent of the physiological antagonism between the two substances is so far limited that a very large fatal dose of strychnia may kill before the chloral has had time to act, or so large must be the dose of chloral hydrate to antagonize an excessive dose of strychnia that there is danger of death from the effects of the chloral hydrate.

5. Chloral hydrate mitigates the effects of a fatal dose of strychnia by depressing the excess of reflex activity excited by that substance, whilst strychnia may mitigate the effects of a fatal dose of chloral hydrate by rousing the activity of the spinal cord; but it does not appear capable of removing the coma produced by the action of chloral hydrate on the brain.

**Aromatized Glycerine and Its Uses.**—The following is Professor Jaccoud's formula—

| | | |
|---|---|---|
| ℞ Glycerine | 40 grams. | |
| Rum or cognac | 10 grams. | |
| Essence of mint | 1 drop. | M. |

This mixture, which is of an agreeable taste, is well tolerated by the stomach, so that after several months of its uninterrupted employment it gives rise neither to satiety nor disgust. The addition of the alcohol has simply in view the modification of the insipid taste of this drug, and to aid in its digestion. Its dose would be quite insufficient as an alcoholic medication. The quantity of glycerine mentioned in the formula is the minimum daily dose, and it may be increased to fifty or sixty grams. This last quantity, however, should not be given except to persons who present no sign of abnormal excitement of the nervous system or of the action of the heart. Moreover, whenever there is any agitation or unusual loquacity, obstinate sleeplessness, or a persistent elevation of temperature (in the absence of any pyretogenic incident) of 0.5°C. in relation to the mean temperature of the period prior to taking the glycerine, it will be an indication that the serviceable dose has been exceeded. Glycerine should be employed for the purpose of stimulating the digestive functions, and saving the waste of tissue during the non-febrile period of phthisis, when cod-liver oil has ceased to be tolerated. The dose of aromatized glycerine should be divided into two or three takings.—*Med. and Surg. Reporter.*

**Enlarged Spleen.**—A Texas correspondent of Gaillard's Medical Journal states that the following drugs have proved most effective in this very common affection: "First, in importance, is, we believe, the iodide of manganese, in one or two grain doses. The tincture of iodine, in ten-drop doses, *ter die*, until its constitutional effects are manifested, is, we think, of great value. A large blister over the affected organ, and repeated as rapidly as possible six or eight times, was of great benefit to the writer; but it is difficult to persuade patients to submit to more than one blister while able to go about and attend to their daily business. The muriate of ammonia in five grain doses, is another valuable remedy. But, with one and all of these, we often fail to find any reduction in the size of the organ or in the tenderness over its site. One remedy that we should have mentioned as often beneficial, is oil of black pepper, in combination with prussiate of iron."

He fails to mention the internal administration of arsenic, and injection of fluid extract of ergot into the organ, as recommended by Hammond.—*Clinical Record.*

**A Resurrection.**—Bucharest papers relate the following incident: A young woman died of small-pox, and, pursuant to police regulations in times of epidemics, the body was interred without loss of time. The girl had been engaged to be married, and, accordingly, the relatives had adorned the body of the deceased with the jewels that had been presented to her. These trinkets aroused the cupidity of three individuals, who resolved to desecrate the tomb. The grave was opened, the body taken out, and the jewels removed. During these procedures one of the robbers was, by his companions, accused of cowardice, and to disprove the assertion he launched a terrible blow at the dead girl. The effect was magical; the supposed corpse assumed the sitting posture and begged for mercy; the robbers, however, fled terror-stricken in hot haste. The girl arose and with trembling steps sought the dwelling-place of the curate, who, after a preliminary scare, finally comprehended the situation, and volunteered to prepare her relations for the news. Joy reigned supreme. In consideration of the service they had rendered the robbers were not prosecuted.—*N. Y. Med. Record.*

**Prizes Worth Striving For.**—The Royal Academy of Medicine of Brussels announces a prize to be awarded in January, 1884, of eight thousand francs ($1,600), for the best explanation of the pathology and therapeutics of the diseases of the nervous centers, especially epilepsy, illustrated by clinical data and experiments. If the essayest is successful in making a decided advance in the therapeutics of such diseases; if, for instance, he discovers a successful treatment for epilepsy—he is to receive, in addition to the sum above stated, a second sum of twenty-five thousand francs ($5,000). This money has been placed in the hands of the Academy by a person who does not wish his name to appear.

In Italy, the Academy of Medicine of Turin offers a prize of twenty thousand lire ($4,000) for the best essay on "The Physiopathology of the Blood." This is the Riberi Prize, and is open to the world, but the essay must be either in the Latin, French or Italian tongue.—*Med. and Surg. Rep.*

**A Case of Prolonged Somnolence** that may serve as a companion-piece to that of the sleeping Hungarian in Pennsylvania, is reported from one of the hospitals of Niederweised, in Germany. The twelve-year-old daughter of an inn-keeper fell into a deep trance in March, 1880, and continued in that condition for the entire remainder of the year. No medicine was given her, and the small quantity of nutriment that was prescribed had to be administered by forcing her mouth open. She had normal sleep at night, but during the day lay wholly motionless, and apparently without sensation or consciousness. At first much emaciated, her appearance subsequently became fresh and healthy. About the beginning of the present year she suddenly recovered her power of speech, and was soon wholly restored in other respects. She is now entirely well. It is also said that during the whole period of her suspended animation she was fully cognizant of everything that took place about her.—*N. Y. Med. Record.*

**Morbus Coxarius Treated by the Physiological Method.**—Dr. J. C. Hutchinson (American Journal of Medical Science, January, 1879), advances the view that to secure immobility of the diseased joint, and to obtain extension of the limb, no apparatus is required. He simply attaches to the sole of the shoe of the sound limb a steel plate corresponding to the sole of the shoe, and attaches to it by two or three upright rods, two or three inches in length, so as to raise the foot from the ground. It is the shoe ordinarily used for the shortened leg. This elevated shoe and a pair of crutches constitute the entire apparatus. Extension of the limb is made by its own weight, also immobility, while with his crutches the patient can have plenty of out door exercise. The doctor's experience warrants the expectation that this plan of managing coxalgia will shorten its duration more decidedly than can be done by the older methods of treatment. The apparatus is simple and inexpensive.—*Detroit Lancet.*

**Ergot in Tuberculosis.**—A writer in the Cincinnati Lancet and Clinic suggests, on theoretical grounds, the use of ergot in the early stages of tuberculosis, with a view to prevent the formation of tubercular deposits. "We know," says he, "that ergot contracts blood vessels. Could it not be possibe that, by giving ergot as soon as we are aware of tubercular matter being formed, it would contract the blood vessels and prevent the exudation and somatic condition of the blood vessels' walls? Even if ergot is given later on in the disease, and deposits have taken place, if we can stop the further progress of the disease, the deposits that have already been formed are likely to undergo fatty or calcareous degeneration, and do no harm. It might be possible that by the action of ergot in conjunction with alcohol and cod-liver oil, it might be of some benefit, if nothing more."

We hope he will try it, and then let us know the result.—*Med. and Surg. Reporter.*

**The Treatment of Erysipelas.**—Dr. W. Thackeray, of this city, (Philadelphia) says, in the Therapeutic Gazette for August, 1881, the idiopathic variety, which also indicates the so-called phlegmonous, has proved in his hands a very tractable disease, and he has witnessed and treated some of the most distressing and violent attacks possible to occur. All that is necessary locally, says he, is to keep the parts affected well covered with a cloth wetted with a saturated solution of sodium chloride, and constitutionally to administer a refrigerant of citrate of magnesia, made extemporaneously, from the sulphate and lemon juice. This plan, with a light farinaceous diet, will cut short the most violent attack, and will leave the patient without the unsightly scars that remain as a sequel to the more energetic course generally adopted.—*Med. and Surg. Rep.*

**Common Bread Poultice.**—Dr. William S. Savory (British Medical Journal, August 9, 1879), says that again and again has he been driven to return to the use of the common bread poultice, by the fact that it fulfilled certain conditions better than any of its rivals. A well-made bread poultice preserves ample moisture and equable warmth, it is everywhere very soft, and adapts itself with singular uniformity to all irregularities of surface. In my experience this homely article more frequently draws from the patient the word comfort than any other form of dressing.—*Detroit Lancet.*

**The Reported Death** of Dr. H. S. Tanner, at Amsterdam, Holland, which gained such wide-spread publicity, was either a sheer fabrication or some other person attempted to personate him in Europe.

The real Dr. Tanner, of fasting fame, is not dead, but is now actively engaged in the practice of his profession, in Corry, Pennsylvania. We received a letter from him a few days ago, in which he says, "I see you have been interviewed as to the story going the rounds of the press that I died in Amsterdam. Well, if you are inquired of further in regard to this matter, you can be very emphatic in branding the story *an Amster*-DAM lie."—*Mobile Tribune.*

**Dangers of Tents.**—At a meeting of a New York Obstetrical Society, Dr. T. A. Emmett said that in his experience dangerous consequences were especially liable to follow the use of tents in nervous and hysterical subjects. He referred to a case that he had reported last winter, in which trouble did not occur until the seventh day, The patient should never be allowed to get out of bed until the next day after the removal of the tent. In spite of all precautions, he always felt, when about to use a tent, that he was endangering his patient's life.—*N. Y. Med. Journal.*

**Purgatives by Hypodermic Injection.**—It has been found (Paris Medical) that hypodermic injections of aloin (the alkaloid of socotrine aloes) will cause purgation when used in doses of one twenty-fifth of a grain. We can thus, by combining apomorphia and aloin, produce an action each way without having to introduce anything into the stomach.—*Western Lancet.*

**Fish Bones in Pharynx.**—It is said that "fish bones lodged in the pharynx are rendered flexible, and are finally broken up by a mixture of hydrochloric acid (four parts) or nitric acid (one part to two hundred and forty parts of water), used as a gargle, the teeth being protected by oil or lard."—*Ex.*

**For a Dinner Pill.**—Fothergill, in London Practitioner. Ipecacuanha forms a portion of a good old-fashioned dinner-pill; and betwixt its direct action upon the gastric mucous membrane and its action on the liver as an hepatic stimulant, it must come into use again before long. A dinner-pill of

| | |
|---|---|
| Pulv. ipecacuan | gr. j, |
| Strychniæ | gr. 1 20, |
| Ol. pip. nig | *m* ij, |
| Pil. al. et myrrh | gr. ijss. |

Every day, will often produce excellent effects. Then arsenic may be taken, as three drops of Fowler's solution after dinner, or in the above pill, substituting the same dose of arsenic for the strychnine.—*Can. Jour. Med. Sci*

**Gonorrhœa.**—The sulpho-carbolate of zinc has been extalled by Dr. W. T. Parker, of Mass., as an injection in gonorrhœa—

| | | |
|---|---|---|
| ℞ | Zinci sulpho-carbolatis | ʒj, |
| | Mucilag. acac | ℥j, |
| | Extract opii aquosi | ℈j, |
| | Aquæ | ℥vj. |

M. Use as injection, night and morning.

Hydrastis still finds advocates. Bartholow recommends a drachm of hydrastia (the alkaloid) to four ounces of mucilage of acacia, and has found no injection so uniformly successful. Phillips prefers an injection made by adding one or two drachms of the tincture to a pint of water, and of this orders a syringeful to be injected up the urethra every half hour for seven or eight hours for two or three days.

In the Bull. Gen de Therapeutique, 1880, Dr. Pasqua reports four cases of gonorrhœa in its early stage treated with a chloral solution—

| | | |
|---|---|---|
| ℞ | Chlorali hydrati | gr. vj, |
| | Aquæ rosæ | fl. ℥j. M. |

Two urethral injections daily were used, the fluid being retained a few minutes in the urethra. Improvement began in four or five days, and the discharge ceased in eight or ten days. No unpleasant sequelæ appeared.—*Med. and Surg. Rep.*

